# 生物芯片技术在肺癌研究中的应用价值

**DOI:** 10.3779/j.issn.1009-3419.2011.05.11

**Published:** 2011-05-20

**Authors:** 珉 朱, 军 于, 文利 周, 向宁 付

**Affiliations:** 1 430030 武汉，华中科技大学同济医学院附属同济医院普胸外科 Tongji Hospital, Tongji Medical College, Huazhong University of Science and Technology, Wuhan 430030, China; 2 430074 武汉，华中科技大学电子科学与技术系 Department of Electronic Science and Technology, Huazhong University of Science and Technology, Wuhan 430074, China; 3 100084 北京，生物芯片北京国家工程研究中心 National Engineering Research Center for Beijing Biochip Technology, Beijing 100084, China

**Keywords:** 肺肿瘤, 生物芯片, 诊断, 预后, Lung neoplasms, Bio-Chip, Diagnosis, Prognosis

## Abstract

肺癌的发生发展是多基因共同参与、多途径相互影响的复杂生物学进程，与多种癌基因、抑癌基因、DNA修复基因等变异或表达异常有关。如何从海量数据中寻找肺癌的发病因素和癌变多阶段演进的分子机制，是目前科研和临床工作者必须面对的问题。生物芯片技术的诞生为人们提供了一种高通量、高效率的肿瘤学研究手段。本文将讨论生物芯片技术在肺癌中的最新研究进展。

## Abstract (en)

Lung cancer is a complicated disease involving many genes and important biologic processes. Genetic variation or aberrant expression of some oncogenes, tumor suppressor genes and DNA repair genes might be related to its incidence and development. How to find out the pathogenic factors of lung cancer and its molecular mechanism of multistage carcinogenesis from massive data is a key question that researchers and clinicians must answer. The bio-chip technology provides a high throughput and efficient method for cancer research. The latest bio-chip progress in lung cancer research is summarized in this minireview.

生物芯片技术是上世纪末生命科学领域取得的最大科学技术进展之一，是将各种生物信息分子如寡核苷酸、基因片段、cDNA片段或多肽、蛋白质等按预先设置的排列高密度固定在固相支持介质上行成微阵列，利用生物分子的特异性亲和反应，如核酸杂交反应、抗原抗体反应等进行各种定性、定量分析的一种技术。其连续化、集成化、自动化，高通量的特点为后基因组时代基因功能研究、新药研发及疾病早期诊断、转归、预后评估等提供了强有力的分析工具。自诞生以来，生物芯片技术发展迅猛，目前已经形成了全球年产值达近百亿美元的销售市场，具有明显的高科技产业化前景。肺癌是临床上常见的恶性肿瘤之一，目前已成为所有癌症中递增速度最快的恶性疾病^[1]^。近年来，尽管肺癌综合治疗技术有了很大进步，但肺癌长期生存率仍只有10%-15%^[1]^。主要原因是肺癌起病隐匿，现阶段仍缺乏有效的筛查和早期诊断方法。目前已证实肺癌的发生发展是多基因共同参与、多途径相互影响的复杂生物学进程，与多种癌基因、抑癌基因、DNA修复基因等变异或表达异常有关^[2]^。如何跳出过去仅仅局限于单基因的肿瘤分子学研究禁锢，从海量数据中寻找肺癌的发病因素和癌变多阶段演进的分子机制，是目前科研和临床工作者必须面对的问题。生物芯片技术的诞生克服了传统检测技术复杂、自动化程度低、检测目的分子数量少、低通量等不足，为人们提供了一种全新的思维方式和微系统形式加速揭开癌症的分子生物学奥秘^[3]^。本文将讨论生物芯片技术在肺癌中的最新研究进展。

## 1 生物芯片分类及应用

生物芯片可分为基因芯片、蛋白芯片、细胞芯片和组织芯片等。世界上第一块生物芯片即美国Affymatrix公司研制的基因芯片。该公司的研发人员于1992年运用半导体照相平板技术，对原位合成制备的DNA芯片作了首次报道。随着化学染料、微电子技术、激光、数据分析技术的更新，目前基因芯片技术得到了迅猛的发展，其应用范围也逐渐扩大。基于基因芯片的比较基因组杂交技术（comparative genome hybridization, CGH）在保留了CGH技术样本量要求低、全基因组快速扫描等优点的同时，解决了其敏感性差、自动化程度低、操作复杂等技术问题，成为支持研究人员通过基因芯片技术准确研究与疾病有关的染色体变化的崭新平台。Broët等^[4]^运用该技术对85例Ⅰb期肺腺癌组织进行了分析，结果发现56.5%的病例存在染色体5p的扩增，与此对应的是此区域端粒酶逆转录酶基因（telomerase reverse transcriptase, TERT）和泛素连接酶基因（S-phase kinase associated protein 2, SKP2）的大量扩增事件。此外，8q24（c-MYC）、11q13（CCND1）及14q13（TTF1）区域也发生了节段性扩增。这些变化与患者是否存在肿瘤复发和预后密切相关，作者认为分析肺癌染色体变化特征对于预测早期肺癌的预后大有帮助。国内虽然在基因芯片技术开发上起步较晚，但目前也具有地中海贫血、肺结核杆菌及其耐药性、乙肝等疾病的检测芯片专利。因为生物体的众多功能最终都要通过蛋白质来实现，故在活性基因所表达的mRNA与蛋白质之间未能显示出直接关系的前提下，基因芯片技术的应用必然会受到一定的限制，蛋白芯片技术也就随之应运而生。蛋白芯片基本原理类似于酶联免疫吸附试验方法，即利用抗原抗体结合的特异性来检测受检标本内的抗原或抗体。通过报告分子如荧光标记的位置、荧光强弱等信号转换成数据，即可以获得有关的生物信息^[5]^。如多肿瘤标志物蛋白芯片检测系统可以对多种肿瘤标志物进行联合检测，从而解决了在临床诊断领域一直困扰人们的难题-如何快速而有效地早期发现肿瘤。表面增强激光解吸电离-飞行时间-质谱（surface enhanced laserdesorp-tion/ionizationtime of flight mass spectrometry, SELDI-TOF-MS）技术，可使吸附在蛋白芯片上的靶蛋白离子化，在电场力的作用下计算出其质量电荷比，与蛋白质数据库配合使用，可检测到经典方法无法测定的低丰度蛋白质。Au等^[6]^利用该技术从21例无吸烟史的肺癌患者组织中筛选出与癌旁正常组织完全不同的新的痕量蛋白标记物，其进一步的纯化和确定将为无吸烟史肺癌的诊断和靶向治疗提供新的途径。在目前提倡肿瘤个体化治疗的背景下，蛋白芯片技术在新辅助化疗敏感与耐药差异蛋白筛查中也具有潜在的临床应用价值^[7]^。细胞芯片和组织芯片技术是近年来基因芯片技术的发展和延伸。细胞芯片可以建立活体的多相反应体系，克服了基因、蛋白芯片仅仅检测生物信息分子是否表达或表达量高低，而无法评估生命最基本单位-细胞整体功能的缺陷^[8]^。在新型药物研发过程中，利用细胞芯片上的靶细胞筛选药物，不仅可以提高药物开发的效率，而且可以实现药物筛选的敏感性、高通量和自动化集成。北京-生物芯片国家工程研究中心利用实时细胞阻抗传感技术，实现了在细胞芯片上对细胞迁移过程的实时监测，为生物体胚胎发育、伤口愈合、肿瘤转移等众多生物过程提供了独特而巧妙的检测方法^[9]^。组织芯片则具有将分子生物学和组织形态学相结合的优势，克服了传统组织分析研究的瓶颈。结合免疫组织化学、原位杂交、原位PCR等技术，可以在基因、蛋白、细胞等不同水平进行形态与功能的研究^[10, 11]^。Fernandes等^[12]^利用组织芯片技术在观察到硫氧还蛋白家族蛋白在不同分化程度的肺癌组织中的差异表达后，阐明了硫氧还蛋白家族蛋白表达与肺癌肿瘤细胞增殖与分化之间的关系，从而为肺癌的发病机制提供了新的分子学基础。陈洪雷等^[13]^以组织芯片为载体，利用量子点技术检测Cav-1、CD147、MMP-2在人肺癌组织中的表达。不仅建立了有效的高通量蛋白表达分析系统，还证实Cav-1在肺癌侵袭转移中的关键作用。

## 2 肺癌机制研究

肿瘤的形成都伴随着DNA甲基化和组蛋白的修饰异常，其中异常甲基化包括基因组的低甲基化（hypomethylation）和抑癌基因启动子CpG岛高度甲基化（hypermethylation）。因检测手段的效率低下，过去人们对DNA甲基化在癌症致病中的认识十分有限。Rauch等^[14]^近期发明了一种基于MBD2b/MBD3L1蛋白复合物对甲基化CpG二核苷酸高亲和力的甲基化高分辨率检测方法——MIRA，在以微矩阵形式实现其高通量检测后可以评估全基因组甲基化。研究者着重探讨了*HOX*基因甲基化与肺癌发病之间的关系。通过对肺癌细胞系A549和正常人支气管上皮NHBE细胞的大规模基因组扫描，将分别位于染色体2、7、12和17的HOXA、HOXB、HOXC和HOXD基因簇进行了甲基化分析。结果提示旁系同源基因HOXA6、HOXB6启动子在A549细胞中发生高度甲基化，而在NHBE细胞中则无甲基化现象。在进一步的Ⅰ期原发肺癌组织中的检测表明，肺腺癌和肺鳞癌有不同的*HOX*基因甲基化谱，后者具有较少程度的*HOX*基因甲基化现象，但HOXA9与Ⅰ期肺鳞癌的发病高度相关，具有较好的早期诊断价值。表皮生长因子受体（epidermal growth factor receptor, EGFR）家族参与多种实体瘤的发生与发展，是抗肿瘤治疗的重要靶点。在非小细胞肺癌（non-small cell lung cancer, NSCLC）中，*EGFR*突变与其磷酸化事件在NSCLC发病中的具体信号机制尚未阐明。VanMeter等^[15]^采用反向蛋白芯片（reverse phase protein microarray, RPPM）技术，结合激光捕获显微切割方法，观察25例NSCLC患者肿瘤组织微环境中EGFR家族酪氨酸激酶结构域突变与其自身和下游信号分子的磷酸化事件。在所研究的6个磷酸化位点中，所有*EGFR*突变型患者肿瘤组织中的Tyr-1148、Tyr-1068磷酸化明显增多，而Tyr-1045、HER2 Tyr-1248、IRS-1 Ser-612以及SMAD Ser-465/467磷酸化则显著减少。研究者在体外*EGFR*突变型NSCLC细胞系的类比实验中观察到了相似的磷酸化事件，从而推测高突变率NSCLC细胞通过Tyr-1148、Tyr-1068磷酸化激活酪氨酸激酶活性，持续地触发PI3K/AKT/mTOR等增殖信号。另一方面，高突变率NSCLC细胞还可同时通过减少Tyr-1045、IRS-1 Ser-612磷酸化及改变HER2异源二聚化途径减少EGFR泛素化，使其失去对转化抑制的能力，最终导致细胞癌变。

## 3 肺癌早期诊断

肺癌的早期诊断一直是人类梦寐以求的目标。年龄、吸烟史、职业暴露史、咳血、体重减轻、肿块大小、肺活量测定等变量在预测早期肺癌上尚难以作出准确的判断。Spira等^[16]^通过基因芯片在吸烟人群中对呼吸道上皮细胞基因表达差异进行分析，可以早期诊断潜在的罹患肺癌患者。研究者甄别出一个含80个基因簇的生物标志物集合，通过评分系统在吸烟者中判断潜在肺癌和未患肺癌的准确性达到了83%，其特异性和敏感性分别为84%和80%。进一步的研究证实该生物标志物集合可以在早期预测Ⅰ期肺癌的敏感性上达到90%，如能结合纤维支气管镜检查进行细胞病理学分析，则可将肺癌早期诊断的敏感性进一步提高到95%。与临床危险因素所组成的模型相比，该临床基因组学模型对预测早期肺癌的敏感性有显著性差异。特别在临床医师无法确定肿块性质的患者当中，运用该基因组学模型对判断是否罹患肺癌具有相当高的准确性^[17]^。Ye等^[18]^通过液相基因芯片技术从125例（37例小细胞肺癌，88例NSCLC）患者的组织中提取DNA进行研究，结果发现p53+EGFR二联突变检测对肺癌诊断的敏感性为50.4%；p53+p16+EGFR三联突变检测则可将敏感性提高到60%；当联合检测p53、p16、视网膜母细胞瘤*Rb*、*EGFR*基因的突变事件时，肺癌的诊断敏感性进一步提高到68%，且四联基因突变检测的特异性和准确性分别为90%和72.3%。Madoz-Gúrpide等^[19]^使用蛋白芯片技术从肺腺癌细胞系A549细胞的1, 760个蛋白片段中筛选出肺癌的潜在蛋白标记物，然后通过质谱分析及利用肺癌患者血清与其产生的抗原抗体反应确定该类蛋白为PGP9.5，而正常健康人对照则无此反应，为肺癌的早期诊断提供了新的候选标志物。

## 4 肺癌靶向治疗

由于药物基因组学的飞速发展，传统化疗药物的分子标志物不断被发现，以其为指导的临床试验不断取得令人惊喜的结果，推动着传统化疗进入个体化靶向治疗的时代。目前，EGFR酪氨酸激酶抑制剂（tyrosine kinase inhibitor, TKI）在肺癌靶向治疗中的应用最为广泛。在一项关于厄洛替尼治疗NSCLC的多中心开放性临床Ⅱ期试验^[20]^中，研究者从264例处于Ⅲb期/Ⅳ期的NSCLC患者肿瘤组织中提取核酸进行基因芯片分析，试图寻找出接受厄洛替尼治疗并从中获益患者的基因表达谱特点。结果提示：临床获益患者的基因谱与无明显效果的患者相比，EGFR、磷酸丝氨酸磷酸酶和Rap鸟苷酸交换因子5三种基因的表达显著增高，且其表达与较长的无进展生存期呈正相关。Seike等^[21]^使用含389个探针的miRNA微阵列芯片对28例非吸烟肺腺癌患者病变组织和正常对照组织比较，结果发现有5种miRNA表达上调，其中miR-21变化最为显著；13种miRNA表达下调，其中miR-486和miR-126^*^下降最为明显。在进一步的研究中，作者分别探讨了EGFR突变型和野生型肺腺癌细胞系H3255和H441与miR-21表达的关系。证实EGFR无论突变与否，如能抑制miR-21表达，H3255和H441均可发生凋亡，为肺腺癌的靶向治疗提供了理论依据^[20]^。Stathmin蛋白家族由于其特有的微管解聚活性，在细胞的增殖和分化及肿瘤发生中有十分重要的作用。抑制Stathmin蛋白的表达已经成为肿瘤基因治疗的新的靶点。Singer等^[22]^通过组织芯片TMA技术分析了117例NSCLC肺癌患者组织中Stathmin蛋白家族Stathmin及Sclip蛋白的表达水平。结果提示在鳞癌中Stathmin高度表达，而腺癌中两者的表达水平均较正常肺组织显著增高，尤其在组织受侵处异常明显。作者随后利用RNA干扰沉默其家族上游调控蛋白FBP-1的表达，发现不仅Stathmin及Sclip蛋白表达下调，且伴随肿瘤细胞的迁移、侵蚀能力下降。此项研究为靶向治疗肺癌提供了新的途径。

## 5 肺癌预后

Landi等^[23]^使用miRNA微阵列芯片对165例腺癌和125例肺鳞癌患者进行miRNA表达分析，试图发现miRNA表达与肺癌患者预后的关系。通过分层分析，发现肺腺癌中暂无明显差异表达的miRNA，但miR-25、miR-34c-5p、miR-191、let-7e、miR-34a五种miRNA的表达对肺鳞癌的生存期具有较高的预测价值。在107例男性早期鳞癌患者中，基于交叉验证监督原则所具有高死亡率风险的62例患者中最终有36例死亡（58.0%），而具有低死亡率风险的45例患者中最终仅有12例死亡（26.7%）。Chen等^[24]^使用DNA芯片技术分析了185例NSCLC患者的病理标本，从672个具有侵袭特点基因中筛查16个与生存有关的基因，发现*DUSP6*、*MMD*、*STAT1*、*ERBB3*和*LCK*为5个高危基因，是患者无复发生存时间的独立预后因子。Yoshizawa等^[25]^利用组织芯片技术分析了300例NSCLC患者AKT信号途径中相关蛋白磷酸化事件与其预后的关系。结果提示eIF4E、AKT、TSC2、mTOR、S6和Erk1/2蛋白分别有39.9%、78.8%、5.1%、46.7%、27.1%和16.6%发生磷酸化，且eIF4E、AKT磷酸化事件无论是独立还是联合评估均预示较短的生存期。在聚类分析中AKT、mTOR、eIF4E、S6磷酸化阳性患者的生存期最短，而无AKT、Erk1/2磷酸化的NSCLC患者有较好的预后，其5年生存率达到了75%，平均生存中位数为9.4年。在多变量分析中，eIF4E磷酸化为判断NSCLC患者生存期的独立危险因子。

## 6 展望

目前，生物芯片在国内外已形成研究与开发的热潮。因其技术尚无标准化平台，各家公司的芯片技术都各具特色，数据采集出口无法统一，由此带来结果评估上的混乱。但另一方面，基于生物个体遗传信息多样性的特点，生物芯片个体化发展亦成为其研发趋势。作为一项可能成为实验室研究或临床普遍采用的潜在技术，生物芯片在样品制备和标记、增加信号检测灵敏度、功能模块高度集成化等方面仍有较大空间改进。相信这项新一代生物技术能为肺癌诊断、新药开发、个体化治疗及预后评估等方面带来革命性的变化。
